# Bowel obstruction due to retained intraperitoneal left ventricular assist device (LVAD) driveline

**DOI:** 10.1186/s13019-018-0738-1

**Published:** 2018-05-21

**Authors:** Daniel Miklin, Ivy Lewis, Howard Lieberman

**Affiliations:** 1 University of Miami Miller School of Medicine, PO Box 016159, Miami, FL 33101 USA; 2 Jackson Memorial Hospital, 1611 N.W. 12th Avenue, Miami, FL 33136 USA; 3 Ryder Trauma Center, Jackson Memorial Hospital, 1800 Northwest 10th Avenue, Miami, FL 33136 USA

**Keywords:** Retained LVAD, Bowel obstruction, Cardiac transplant

## Abstract

**Background:**

Left ventricular assist devices (LVAD) provide a lifesaving bridge to cardiac transplant. Utilization of these devices is increasing in the United States. When a patient undergoes cardiac transplant, the left ventricular device is surgically removed and the driveline is extracted or left tunneled in the subcutaneous tissue. Our group encountered a rare and previously unreported complication of this device: intraperitoneal infiltration of a retained driveline after cardiac transplant causing a small bowel obstruction.

**Case presentation:**

A 62 year old male with a past medical history of non-ischemic cardiomyopathy induced heart failure, status post bridging left ventricular assist device and orthotopic heart transplant presented with abdominal distention, tenderness, and leukocytosis six days post-transplant. CT abdomen and pelvis revealed dilated loops of bowel, air-fluid levels and a transition point in the proximal small bowel. The patient was diagnosed with small bowel obstruction and taken for exploratory laparotomy. He was found to have a retained intraabdominal LVAD driveline strangulating a loop of small bowel in the left upper quadrant. The driveline was removed and the section of bowel released with return of perfusion.

**Conclusions:**

We had encountered a rare complication of retained left ventricular assist device driveline after cardiac transplant: inadvertent penetration into the peritoneal cavity resulting in strangulation of small bowel. This complication, though uncommon, provides substantial risk to patients previously treated with left ventricular assist devices. Meticulous care must be taken to ensure proper device insertion and extraction, as well as consideration of this etiology when patients present with bowel obstruction after cardiac transplant.

## Background

Left ventricular assist devices provide lifesaving therapy to patients with advanced heart failure. However, implantation and continued use of these devices can lead to major medical and surgical complications. Infection, bleeding, thromboembolism and device malfunction are amongst the most common complications in patients with LVADs, however, there have also been reports of less common complications including bowel obstruction, pancreatitis, cholecystitis and abdominal wall defects [1, 2]. Many of these complications are thought to be related to issues with placement of the LVAD or driveline. However, there have been no cases previously reported in the literature of bowel obstruction secondary to retained LVAD driveline after cardiac transplant. Here, we report a unique case of a patient previously on LVAD therapy who presented post-operatively with small bowel obstruction due to retention of an intraperitoneal LVAD driveline.

## Case presentation

**Fig. 1:**
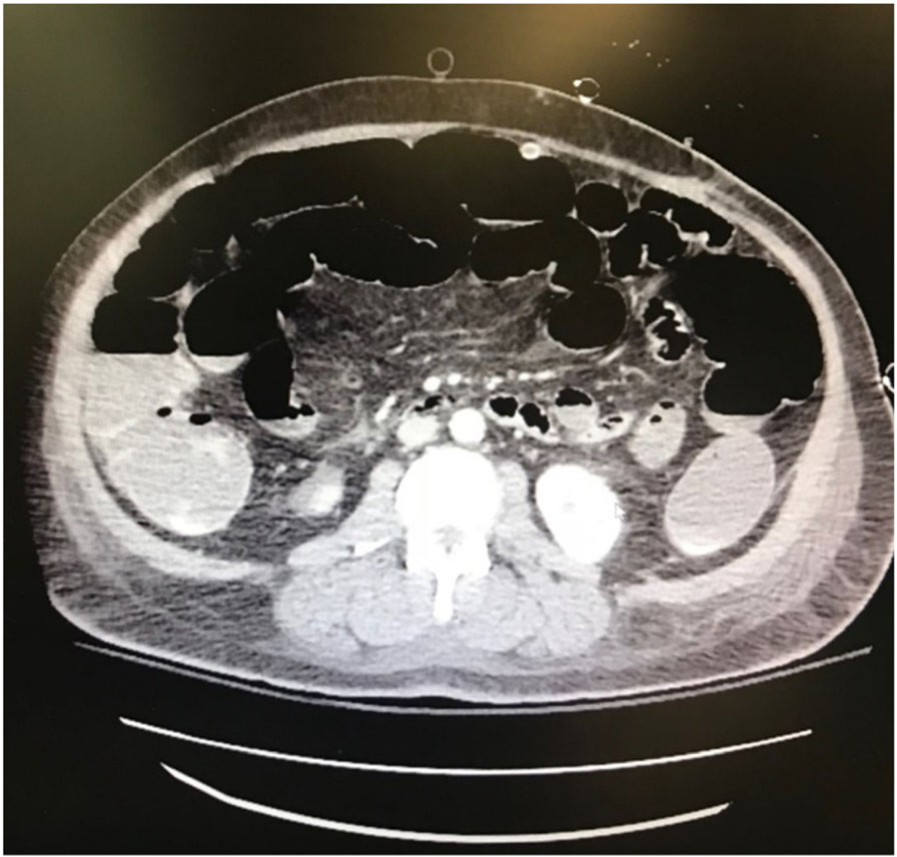
CT Abdomen revealing air-fluid levels, with dilation of multiple loops of bowels, as well as decompressed loops concerning for small bowel obstruction

**Fig. 2:**
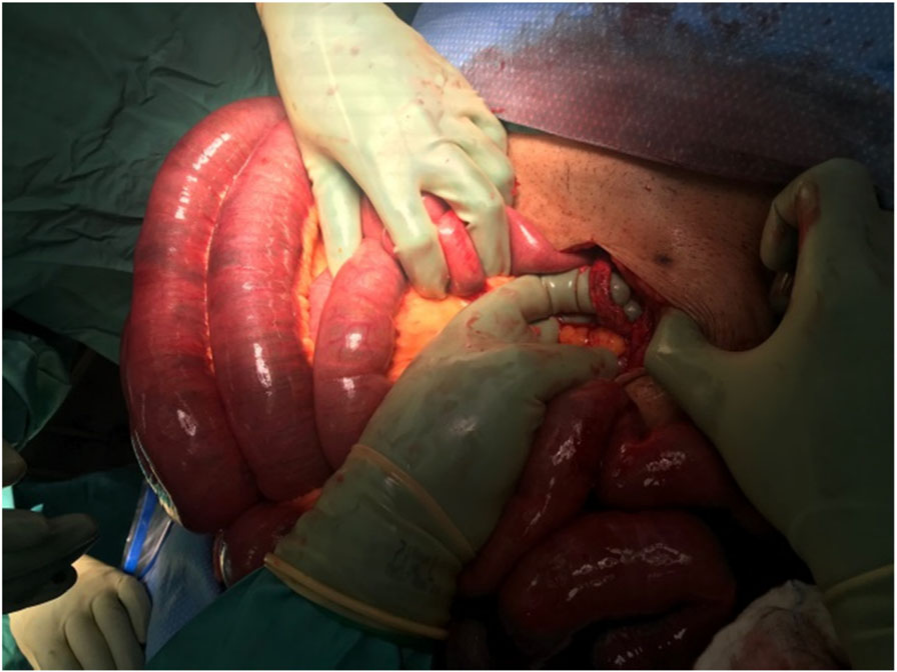
Intraoperative photo revealing intraperitoneal LVAD driveline with small bowel looping behind it

**Fig. 3:**
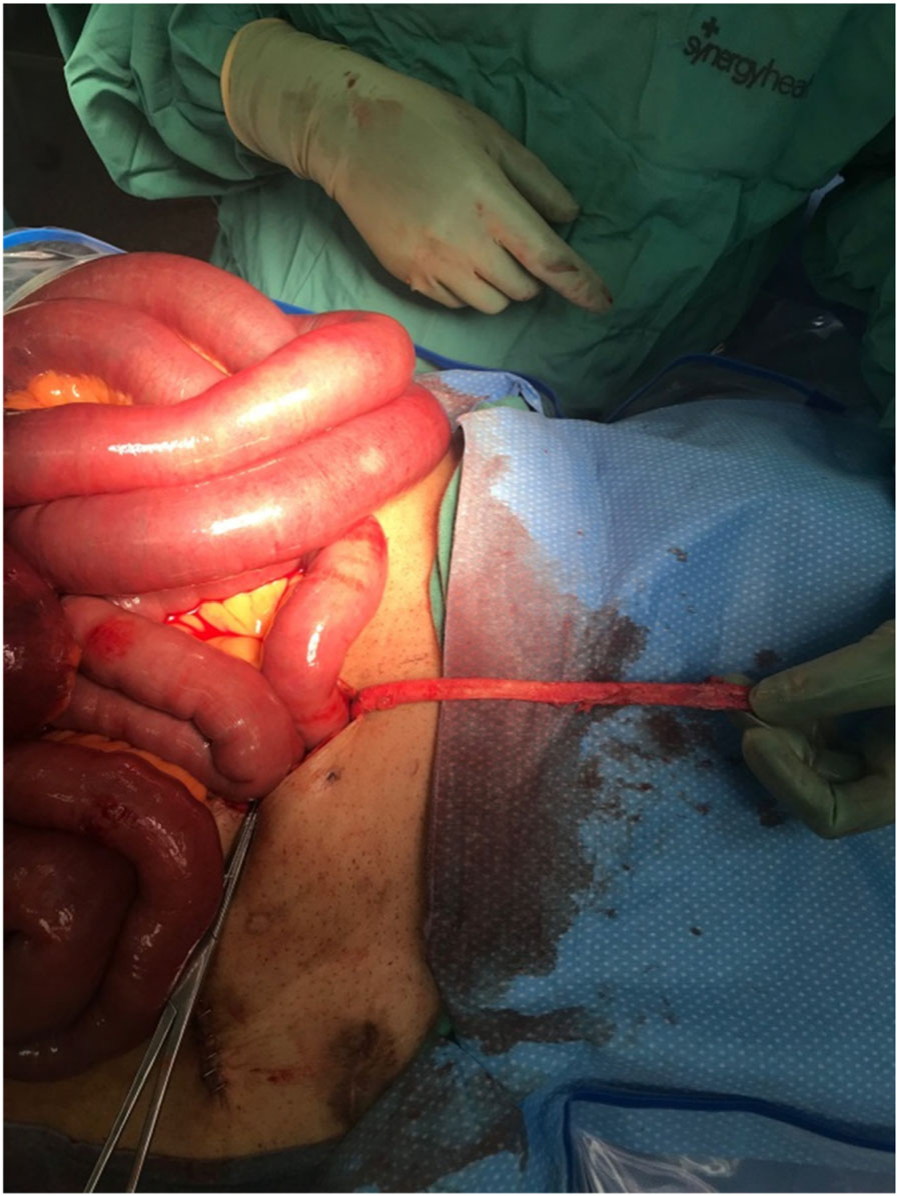
Retained intraperitoneal LVAD driveline

**Fig. 4:**
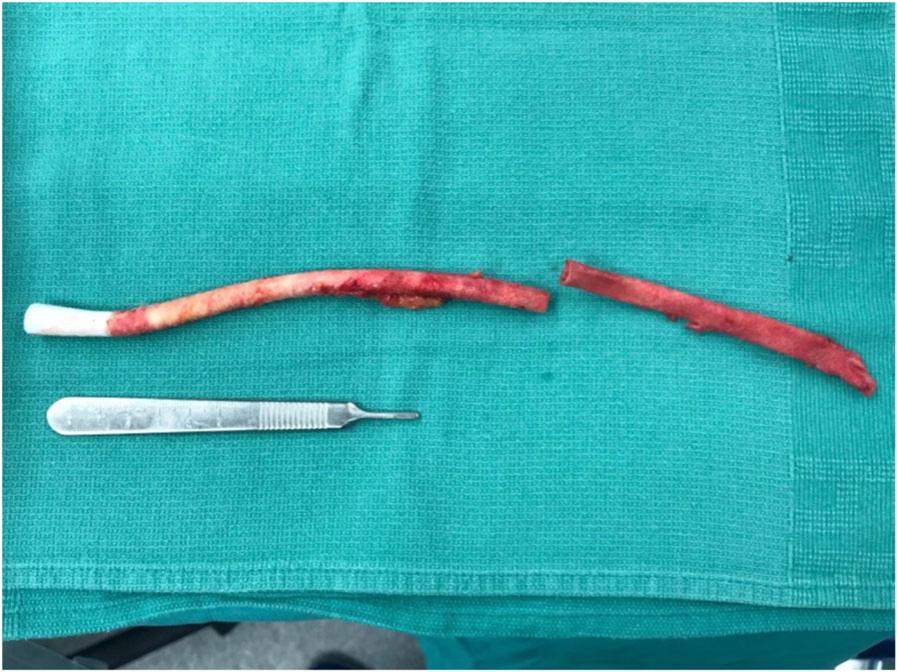
Extracted LVAD driveline in two portions. Scalpel handle for scale

A 62 year old male with a past medical history of non-ischemic cardiomyopathy resulting in heart failure, status post bridging left ventricular assist device underwent orthotopic heart transplant. While recovering in the surgical ICU he developed worsening abdominal distention, tenderness, and leukocytosis, prompting evaluation by the general surgery team 6 days post-transplant. The patient had not passed bowel movements or flatus since his surgery and had not responded to administered enemas or oral metoclopramide for presumed post-op ileus. CT of the abdomen and pelvis was performed which revealed multiple dilated loops of bowel with air-fluid levels and transition point at the proximal small bowel in the left upper quadrant, concerning for obstruction (Fig. 1). Based on these findings as well as the fact that the patient had no prior abdominal surgeries, making bowel obstruction secondary to adhesions less likely, the patient was subsequently taken to the operating room for exploratory laparotomy. In the operating room, the bowel was found to be diffusely dilated without any areas of apparent ischemia. A portion of small bowel was found to be adhered to the anterior abdominal wall in the left upper quadrant. Upon further exploration, a retained LVAD driveline was found to be tunneled into the peritoneal cavity, strangulating a loop of small bowel in the left upper quadrant (Figs. 2 and 3). The driveline was freed from the abdominal wall and removed, and the affected loop of bowel was released (Fig. 4). It was found to be viable after a few minutes of reperfusion. The small bowel was examined in its entirety starting from the Ligament of Trietz distally to the cecum and found to be dilated but grossly normal. A small area of serosal tear at the point of adhesion to the driveline was repaired primarily. The bowel was then returned to the abdominal cavity and the fascia and skin closed with running sutures and skin staples.

## Discussion and conclusions

As the population of heart failure patients increases and transplant volumes remain the same, LVAD therapy is becoming a mainstay of treatment of end-stage heart failure in the US [3]. These devices were initially developed as a bridge to transplant, but indications have been expanded to include short term therapy in patients with cardiogenic shock, or long-term “destination therapy” for end-stage patients who are not eligible for transplant [4]. Commonly known complications of LVAD therapy include risk of bleeding, infection, pump thrombosis, device malfunction and stroke [3]. Less often reported are the non-cardiac surgical complications for LVADs. These may include respiratory failure, abdominal infections and wall defects, intestinal ischemia, obstructive complications, perforated viscus, gastrointestinal bleeding, diaphragmatic defects, cholecystitis, and pancreatitis [1, 2, 5]. Improvements to technology between first and second generation LVADs, as well as modifications towards device placement and driveline tunneling has reduced the incidence of infection, which is by far the most common complication of such [6–8]. Current protocol for LVAD placement includes creating a pocket in the pre-peritoneal space, with the driveline being tunneled under the subcutaneous tissue, above the fascia, and subsequently back out through the skin in the right upper quadrant. Proper tunneling technique is crucial in limiting driveline infections and ensuring proper placement of the device.

Our patient presented 6 days post-orthotopic heart transplant after being previously maintained by a HeartMate II LVAD (Thoratec, Pleasanton, CA). The transplant surgery was successful and the patient was recovering well until he presented with signs and symptoms of bowel obstruction. The patient had no history of prior abdominal surgery, hernias, and had no increased risk for gastrointestinal malignancy, thus the etiology of his SBO was unclear. Due to non-resolution of symptoms, persistent abdominal distention and tenderness, bowel obstruction seen on CT scan, and major risk of morbidity/mortality if a true SBO is present and untreated, the decision was made to undergo exploratory laparotomy. Laparotomy revealed a retained intraperitoneal LVAD driveline that was incarcerating a loop of small bowel in the left upper quadrant, resulting in mechanical obstruction.

This case highlights the importance of proper LVAD driveline placement and awareness of possible rare general surgical complications. Non-operative management of this patient did not resolve his obstruction, and had exploratory laparotomy not been performed the patient could have suffered major morbidity or mortality ranging from bowel ischemia to death. There have been case studies promoting retention of LVAD drivelines after device explantation in order to limit the morbidity of additional surgeries, however the data is limited [9]. Moving forward, we propose that all LVAD explantations should include a device component assessment to ensure all device parts have been removed and are intact, as well as keen post-op observation for retained components and possible related complications. Retention of intraperitoneal LVAD drivelines should be considered in the case of bowel obstructions after cardiac transplant with unknown etiology.

## Abbreviations

LVAD: Left ventricular assist device
SBO: Small bowel obstruction
